# Treatment with surfactants enables quantification of translational activity by O-propargyl-puromycin labelling in yeast

**DOI:** 10.1186/s12866-021-02185-3

**Published:** 2021-04-20

**Authors:** Jennifer Staudacher, Corinna Rebnegger, Brigitte Gasser

**Affiliations:** 1 Christian Doppler Laboratory for Growth-decoupled Protein Production in Yeast, Department of Biotechnology, BOKU University of Natural Resources and Life Sciences, Vienna, Austria; 2 Institute of Microbiology and Microbial Biotechnology, Department of Biotechnology, BOKU University of Natural Resources and Life Sciences, Muthgasse 18, 1190 Vienna, Austria

**Keywords:** Global translation activity, *Komagataella phaffii* (*Pichia pastoris*), O-propargyl-puromycin (OPP), Surfactant, Stress response

## Abstract

**Background:**

Translation is an important point of regulation in protein synthesis. However, there is a limited number of methods available to measure global translation activity in yeast. Recently, O-propargyl-puromycin (OPP) labelling has been established for mammalian cells, but unmodified yeasts are unsusceptible to puromycin.

**Results:**

We could increase susceptibility by using a *Komagataella phaffii* strain with an impaired ergosterol pathway (*erg6*Δ), but translation measurements are restricted to this strain background, which displayed growth deficits. Using surfactants, specifically Imipramine, instead, proved to be more advantageous and circumvents previous restrictions. Imipramine-supplemented OPP-labelling with subsequent flow cytometry analysis, enabled us to distinguish actively translating cells from negative controls, and to clearly quantify differences in translation activities in different strains and growth conditions. Specifically, we investigated *K. phaffii* at different growth rates, verified that methanol feeding alters translation activity, and analysed global translation in strains with genetically modified stress response pathways.

**Conclusions:**

We set up a simple protocol to measure global translation activity in yeast on a single cell basis. The use of surfactants poses a practical and non-invasive alternative to the commonly used ergosterol pathway impaired strains and thus impacts a wide range of applications where increased drug and dye uptake is needed.

**Supplementary Information:**

The online version contains supplementary material available at 10.1186/s12866-021-02185-3.

## Background

Protein synthesis is regulated at several cellular levels, mainly by transcriptional control of gene expression and at the different steps of mRNA translation. Interestingly, there is only a limited number of established methods available to measure changes in global translation as transcriptional control is often focused on in literature.

The most traditional translation measurement methods are based on addition of labelled amino acids or amino acid analogues to the media and measuring their abundance in the newly synthesized proteins. However, such amino acids are expensive or difficult to handle and, importantly, their uptake is reduced when compared to canonical amino acids [1]. Additionally, evidence suggests that yeasts preferentially synthesize some amino acids rather than taking them up, even if present in high abundance [2]. Also, for many industrial applications synthetic minimal media lacking amino acids is used, which would not be compatible with these types of measurements. Lastly, the results may be further biased by the frequency of the labelled amino acid occurring in the proteome [1, 3].

Another well-known method is polysome profiling where free RNA, ribosomal subunits, monosomes and polysomes are separated by sucrose gradient centrifugation. Upon RNA isolation from each fraction, ratios are determined by quantitative PCR, cDNA microarrays or RNA-seq. This method has several drawbacks such as a requirement for specialised equipment, it is labour intensive and large quantities of starting material are needed. Besides, rather than measuring translation activity itself, this method only allows for indirect quantification through ribosome occupancy on transcripts [4].

A fairly recent method that does not suffer from these pitfalls makes use of the antibiotic puromycin to measure translation. Puromycin is similar in structure to aminoacyl tRNAs and is therefore incorporated into the nascent polypeptide chain. Once the molecule binds to the A-site of actively translating ribosomes, translation is terminated and all polypeptides which were actively translated, carry a puromycin label [5, 6]. While puromycin has been used for years to study translation in vitro [7], approaches for in vivo measurements were developed more recently and have been further improved since then [8, 9]. To simplify and improve the labelling, puromycin was modified with an additional terminal alkyl group, resulting in O-propargyl-puromycin (OPP). This additional group can be used for a click chemistry reaction to label OPP, for example, with a fluorophore, allowing for analysis in flow cytometry [10–12]. So far, OPP has been successfully used to measure global translational activity in vivo in different mammalian cells and also mammalian tissue [13–15], but not in yeasts.

It was long believed that yeast shows little uptake of puromycin and are also insensitive to its effects, with the only applicability being in spheroplasts [16, 17]. However, in recent years it was found that intact *Saccharomyces cerevisiae* indeed showed growth inhibition when treated with puromycin, just at much higher concentrations than those necessary for mammalian cells [18, 19]. Approaches aimed at increasing the susceptibility of *S. cerevisiae* to puromycin focused on disturbing cell membrane integrity, either by knocking out a component of the ergosterol pathway, or by targeting the pleitropic drug response [19, 20]. Using such engineered strains (EPP: *erg6*Δ, *pdr1*Δ and *pdr3*Δ) made an in vivo incorporation of puromycin into *S. cerevisiae* proteins possible [20]. However, no such mutants are available for the methylotrophic yeast *Komagataella phaffii* (syn. *Pichia pastoris*), an important industrial protein producer [21]. Furthermore, the *S. cerevisiae* EPP strain showed growth defects and could not be transformed by standard protocols [20]. Thus it is not clear if such drug susceptible mutant cells would be suitable to measure changes in translational activity under conditions relevant for recombinant protein production. Therefore, we set out to establish a method to quantify protein synthesis in *K. phaffii*, which is widely applicable to different strains and conditions.

We propose OPP-labelling in combination with surfactant treatment to increase susceptibility as a method to successfully and reliably measure global translation activity in yeast. We confirmed the applicability of the method by investigating different growth conditions and genetically engineered strains of *K. phaffii*.

## Results and discussion

### Disturbing the sterol synthesis pathway increases susceptibility of *K. phaffii* to puromycin

Yeasts are known to be susceptible only to very high doses of puromycin, but it has been shown in a variety of organisms that usage of extreme doses has distinct effects on cell physiology and translation [6, 9, 20, 22]. Thus, to be able to use OPP-based assays, susceptibility of *K. phaffii* to puromycin had to be determined.

First, the minimal inhibitory and the minimal microbicidal concentrations of puromycin were tested. *K. phaffii* strains were incubated in the presence of 0.01–4.25 g L^− 1^ puromycin in YPD and the presence or absence of growth was recorded after 48 h. These experiments showed that 2.12 g L^− 1^ of puromycin was enough to inhibit growth, while 4.25 g L^− 1^ was needed to reduce the amount of colony forming units (Supplementary Fig. S1). In contrast to this, growth of *S. cerevisiae* was inhibited only at > 8 mM or 4.4 g L^− 1^ [19], therefore *K. phaffii* appears to be more susceptible to this antibiotic. Nevertheless, such high puromycin concentrations are physiologically and economically unfavorable, thus increasing susceptibility of *K. phaffii* was a necessary step to make OPP-labelling feasible.

As a first approach, an *erg6*Δ strain was generated. Erg6 is involved in yeast membrane integrity and disruptions of this gene was shown to increase drug susceptibility in *S. cerevisiae* [20, 23–25]. *S. cerevisiae* puromycin susceptibility was also increased by creating knockouts of the two transcription factors involved in pleiotropic drug response, *PDR1* and *PDR3* [19, 20], however, no homologs for either of these genes were found in the available *K. phaffii* genome sequences [26].

**Fig. 1:**
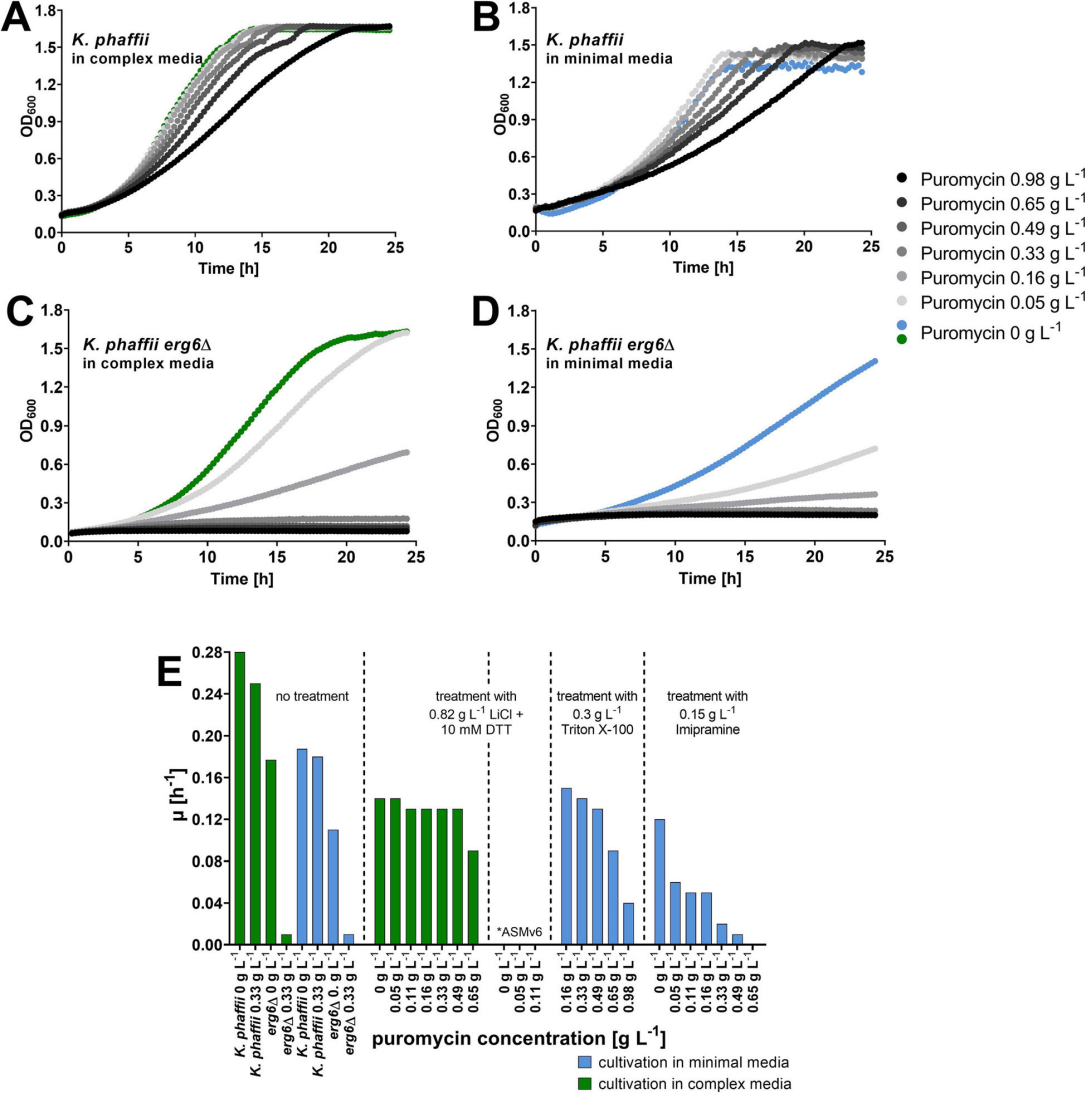
Characterization of *K. phaffii* puromycin sensitivity. For the growth curves, OD_600_ was measured every 15 min in a microplate reader for 24 h at 30 °C and 550 rpm. All growth curves were measured in biological triplicates. The lines represent average values. As controls, untreated cells were included on each assay plate. **a-d** *K. phaffii* CBS2612 (**a** and **b**) and the *erg6*Δ mutant (CBS7435 in **c** and CBS2612 in **d**) were cultivated in complex media YPD (YP + 2% glucose; **a** and **c**), and minimal media (ASMv6 + 2% glucose; **b** and **d**), respectively, with different concentrations of puromycin. In **a-d**, green symbols depict the control without puromycin in complex media, blue symbols are the control in minimal media. **e** μ derived from growth curves of *K. phaffii* CBS2612 cultivations, showing puromycin susceptibility when treated with different surfactants. Blue bars show the results obtained in complex media, while green bars show the results in minimal media

*K. phaffii erg6*Δ showed increased susceptibility to puromycin compared to wildtype *K. phaffii* (Fig. 1a-d). Growth was monitored in complex and minimal liquid media. In the *erg6*Δ strains, growth was strongly reduced with 0.16 g L^− 1^ of puromycin and completely inhibited with 0.33 g L^− 1^ (Fig. 1c-d) which is a reduction of approximately 10-fold compared to the wildtype. Similar levels of sensitivity in the 500 μM range were reported for *S. cerevisiae* EPP [20], therefore, translation activity assays with OPP should be possible with *K. phaffii erg6*Δ. However, the knockout strain showed greatly reduced growth rates in shake flask cultivations at 25 °C in YPD, with the wild type showing μ = 0.28 h^− 1^ and the *erg6*Δ only μ = 0.18 h^− 1^. This effect was even more pronounced in minimal media.

Interestingly, growth defects were also reported for *S. cerevisiae erg6*Δ [23, 25]. Considering, translation activity is correlated to growth rate, using the *erg6*Δ knockout does not appear to be the optimal choice for our purposes.

### Treatment with surfactants also increases susceptibility to puromycin

A potentially less invasive alternative is treatment with chemicals altering membrane fluidity and thereby permeability. Surfactants are believed to increase membrane fluidity by binding to the outer layer of the cell membrane. This in turn results in pore formations, increased fluidity and leakage. Elevated surfactant concentrations lead to leakage of larger molecules and at even higher surfactant concentrations the bilayer starts dissolving completely [27]. Based on evaluation of literature [28–30] two surfactants were chosen as main focus: Triton X-100 and Imipramine (Imp). While Triton X-100 is a commonly used surfactant, Imipramine is better known for its use as human antidepressant. Due to its amphiphilic character it can increase lipid bilayer fluidity until a certain concentration, before it solubilizes the bilayer in vitro [28]. Additionally, a combination of LiCl and dithiothreitol (DTT) was tested, which is commonly used for chemical transformation of *K. phaffii* and should increase membrane permeability as well [31]. Furthermore, PEG4000, Pluronic® PE 6100 and Tween®20 were chosen, as addition of antifoaming agents was reported to increase protein secretion in *K. phaffii* by possibly increasing membrane leakage [32].

Effects of surfactant addition were monitored by combining 5(6)-carboxy-2′,7′-dichlorofluorescein diacetate (5-CFDA) and propidium iodide (PI) stainings. The dye 5-CFDA is known to be cleaved by cytosolic esterases after cell uptake, resulting in a cellular fluorescence signal indicating metabolic activity. PI, a widely used DNA intercalating dye, only enters cells with compromised membrane integrity and is often used to determine cell viability. The flow cytometry results showed treatment with the three antifoam agents, PEG4000, Pluronic® PE 6100 and Tween®20 decreased or did not change the obtained fluorescence signals in comparison to untreated cells (data not shown). At the same time, treatment with high LiCl concentrations resulted in an additional debris peak and decreased cell viability (Supplementary Fig. S2). However, treatment with Imipramine, LiCl/DTT, or Triton X-100 increased the 5-CFDA signal, implying increased molecule uptake by the cells, while cells mostly stayed viable when treated with the concentrations chosen for further testing, as shown by the absence of PI staining (Supplementary Fig. S2 and S3). Therefore, Imipramine, LiCl/DTT, and Triton X-100 were chosen to be tested in further experiments.

One can speculate that proper puromycin intake affects the cells by complete growth inhibition, hence optimal treatment conditions and puromycin concentrations were subsequently determined with growth curve experiments. In this setup, for all of the used surfactants, treated cells showed improved puromycin uptake compared to untreated cells, albeit to a different extent. In cells treated with 0.3 g L^− 1^ Triton X-100 still more than 0.65 g L^− 1^ of puromycin was needed to inhibit growth (Fig. 1e). The combination of 0.82 g L^− 1^ LiCl and 10 mM DTT increased drug uptake in complex media, but cultivation of the treated cells in minimal media was not possible. On the other hand, 0.33 g L^− 1^ (corresponding to 0.6 mM) puromycin appeared to inhibit growth sufficiently in combination with 0.15 g L^− 1^ Imipramine in minimal media. At the same time only 10% of cells were PI-positive, meaning this treatment also did not lead to severe cell death (Fig. 1e, Supplementary Fig. S3 and S4). With Imipramine treatment, the required puromycin concentration is as low as the one needed for inhibiting growth in the *erg6*Δ strain. Thus, similar puromycin susceptibility can be achieved, with the additional advantage that surfactant addition is done directly to the translation activity assay. This means cell growth and production phases remain unaffected which poses a great advantage compared to using a mutant strain. As *erg6*Δ has been implicated with increased uptake of several dyes and drugs that were believed to be not applicable in yeast before [25, 33], increasing membrane fluidity by chemical means could become general practice eliminating the need for such knockout strains. Thus, we could identify a less invasive and simple alternative to increase drug uptake in *K. phaffii*.

### OPP-labelling is possible in untreated *K. phaffii*, but treatment with high concentrations of imipramine increases signal intensity and resolution

OPP-labelling in *K. phaffii* was evaluated in cells and conditions where different translational activities were expected according to literature. Cell growth and translation activity are inherently connected with higher translation activity occurring in faster growing cells [34]. Therefore, we performed OPP-labelling in *K. phaffii* cells growing near their maximum specific growth rate (excess glucose) and slower growing cells (glucose-limited). The glucose-limited conditions were created by using a commercially available kit containing a polysaccharide and a corresponding glucose-releasing enzyme. The slow glucose release rate, which is dependent on the amount of added enzyme, mimics a fed-batch with constant feed and therefore makes sampling at different sub-maximal growth rates possible [35]. Additionally, a no-translation control was included, for which the cultures were supplemented with hygromycin. This antibiotic is known to inhibit translation elongation by binding to the small ribosomal subunit [36].

**Fig. 2:**
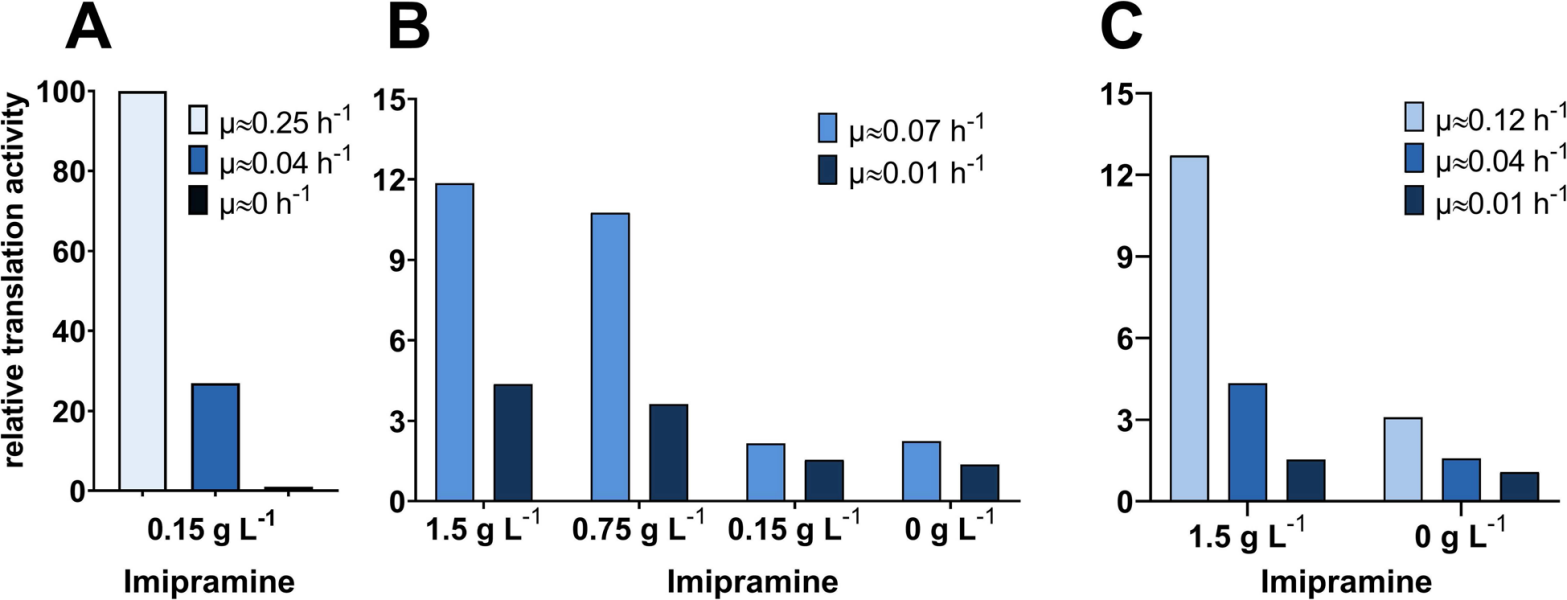
Relative translation activity of *K. phaffii* CBS2612 at different specific growth rates measured by OPP-labelling. All cultivations were done in minimal media, either in glucose-excess (ASMv6 with 2% glucose) or in glucose-limited conditions (ASMv6 containing a polysaccharide and a glucose-releasing enzyme). In the glucose-limited conditions a controlled decrease of growth rate over time is taking place, therefore incubation time determined the obtained growth rates. Cells were treated with Imipramine and 0.3 g L^− 1^ OPP, click labelled with AF488 and the fluorescence signal measured with flow cytometry. **a** OPP-assay of cells treated with 0.15 g L^− 1^ Imipramine. Cells at μ = 0 were cultivated in excess glucose with addition of 20 g L^− 1^ hygromycin to inhibit translation activity. The shown fluorescence signals were subtracted by a no-OPP control and are relative to the signal measured in glucose-excess conditions, which was set to 100. **b** OPP assay of cells treated with different Imipramine concentrations. **c** OPP assay of cells treated with 1.5 g L^− 1^ or no Imipramine. The values shown in **b**) and **c**) consist of the received fluorescence signal relative to the respective no-OPP control and therefore are presented as fold change

After cultivation, the cells were incubated with 0.15 g L^− 1^ Imipramine and 0.30 g L^− 1^ (corresponding to 0.6 mM) OPP. The OPP was, subsequently, conjugated with the fluorophore AF488 using Click chemistry, and the obtained fluorescence was measured in a flow cytometer. The measured differences in fluorescence signal indeed reflected the expected differences in translation activity (Fig. 2a). Fluorescence levels of cells treated with 20 g L^− 1^ hygromycin, the no-translation control, were only marginally higher than background levels. At the same time, cells at a slow growth rate showed greatly reduced translation activity compared to cells growing at maximum speed. Interestingly, we found that the differences in translational activity were visible in cells treated with either 0.15 g L^− 1^ or even no Imipramine during the OPP-labelling assay, but at smaller growth rate differences the received signal differences became difficult to distinguish (Fig. 2b). Therefore, further experiments were needed.

Considering the effect of this surfactant on membrane fluidity, treatment should increase OPP uptake. Thus, treatment with higher concentrations of Imipramine was tested, resulting in greatly increased signal strength and resolution (Fig. 2b). While the differentiation of large changes in translation activity appeared to be possible without addition of Imipramine, smaller differences were only visible and distinguishable from the background noise with higher concentrations. At 1.5 g L^− 1^ Imipramine, even small differences were clearly visible in the fluorescence signals, enabling good resolution of translation activities also at rather slow growth rates (Fig. 2c).

It should be mentioned that incubation with 1.5 g L^− 1^ Imipramine reduced colony forming units by > 99.9% and the cells were PI-positive, while growth was only slightly impaired at concentrations below 0.15 g L^− 1^. However, in the established protocol, incubation with Imipramine and OPP is done at the same time and cells are immediately fixed afterwards. Hence, the measurement represents a snapshot of the global translation activity present at the start of the incubation which is unrelated to the later fate of the cells.

We concluded that even though OPP-labelling is possible in untreated *K. phaffii* with our protocol, additional incubation with 1.5 g L^− 1^ Imipramine results in a significant increase of signal strength and resolution. Therefore this treatment was used in all following assays.

As the method is based on single cell analysis, translation activity can also be correlated to cell size. Interestingly, cells got smaller with decreasing growth rate (lower FSC signal at μ = 0.04 h^− 1^ compared to μ = 0.12 h^− 1^) but the activity of translation per cell volume stayed the same. This could be explained by a decrease in size of other organelles and an increased ratio of cytosol compared to overall cell size. Only at the lowest growth rate setpoint of 0.01 h^− 1^, the translation activity relative to cell size was decreasing as well (Supplementary Fig. S5).

### OPP-labelling shows high translation activity upon methanol feeding

**Fig. 3:**
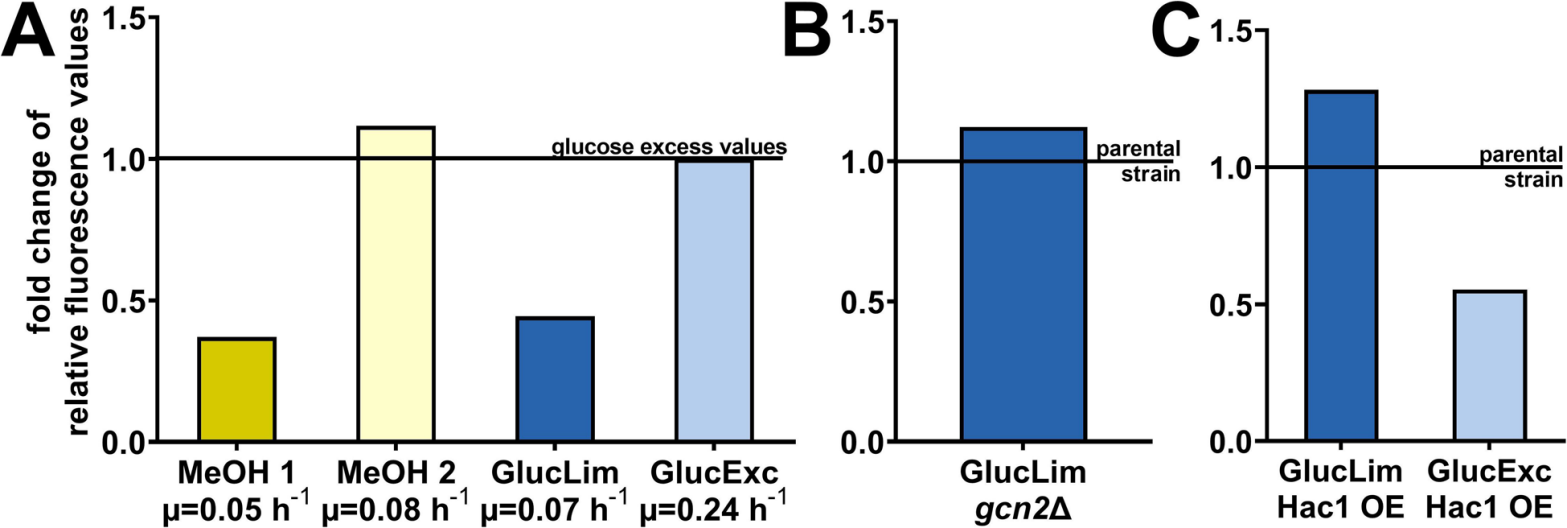
OPP-labelling assays can differentiate conditions and strains with altered translational activity. Assays were done using 0.3 g L^− 1^ OPP and 1.5 g L^−1^Imipramine. GlucLim denotes cultivation under limited glucose and GlucExc denotes cultivation under excess glucose, both in minimal media (ASMv6). All shown values were subtracted by the corresponding no-OPP control. Growth rates were determined by OD_600_ measurements in triplicate 1.5 h before and after sampling took place. **a** Relative translation activities of *K. phaffii* CBS2612 cells grown on different carbon sources. Growth rates were measured by OD_600_. In the MeOH 1 measurement, cells received 1% methanol 3 h before the assay. In MeOH 2, 1% methanol was given 3 h and another time 1.5 h before the sampling. Shown values were normalised to the glucose-excess condition fluorescence signals. All samples were measured in quadruplicate and a no-OPP control was made for each feed strategy in duplicate. **b** Translation activity of *K. phaffii gcn2*Δ relative to its unmodified parent. Cultivation was done in minimal media with glucose-limit to obtain equal growth rates. **c** Translation activities of GS115 P_GAP_His + HAC1 relative to the empty vector control, GS115His+, under different cultivation conditions. Measurements were done in quadruplicate

As a methylotrophic yeast, *K. phaffii* can use methanol as sole carbon and energy source [37]. Methanol metabolism requires additional metabolic enzymes and a higher abundance of peroxisomes. It was found that the protein content of *K. phaffii* biomass is higher when the cells are grown on methanol compared to glucose [38], however, it is not known if this is associated with higher global translational activity. Thus, OPP-labelling was done for cells that received feeding with methanol shots, as routinely done during recombinant protein production, and compared to glucose grown cells. As can be seen in Fig. 3a, methanol shots at different timepoints before measurement resulted in changes of global translation activities, which could be clearly differentiated by OPP-labelling.

Moreover, the measured global translation activity was similar to the cells grown in excess glucose, even though methanol fed cells showed a significantly lower growth rate. Our data nicely resemble the effects seen before with polysome profiling in the commercial *K. phaffii* strain X-33 [39]. Methanol grown cells indeed have a higher translational capacity, which facilitates high level synthesis of methanol utilization enzymes upon their induction.

### Increased translation activity caused by knockout of eIF2 kinase Gcn2 is clearly detectable by OPP-labelling

Finally, translational activity was assessed in two strains with genetically modified stress response pathways to further verify the suitability of the OPP-labelling assay. First, *K. phaffii gcn2*Δ was generated, which carries a knockout of protein kinase Gcn2. Gcn2 inactivates translation initiation factor eIF2, by phosphorylation of its alpha subunit upon stimulation by uncharged tRNAs. This results in reduction of global protein synthesis, but also leads to de-repression of Gcn4-dependent genes in *S. cerevisiae* [40, 41]. *K. phaffii gcn2*Δ and its parent strain were cultivated at equal growth rates, by using glucose-limited conditions, ensuring that the resulting differences derived from the genetic modification rather than from different growth capacities of the strains as described for *S. cerevisiae* [42]. In the OPP-assay a clear difference in translation activity between the two strains was visible (Fig. 3b). Corresponding to literature for glucose limited conditions [43], also *K. phaffii gcn2*Δ showed higher translation activity due to a lack of translational repression.

### Induction of the Unfolded Protein Response (UPR) affects global translation activity

The second target chosen was the transcription factor Hac1, a master regulator of the UPR which can lead to a considerable shift in the global proteome [44]. In mammals, UPR induction leads to attenuation and reprogramming of bulk protein synthesis in order to reduce protein folding load and re-establish homeostasis [45]. Although many aspects of the UPR are conserved across evolution, translational attenuation was so far not reported in yeast. However, overexpression of Hac1 increased recombinant protein secretion in several yeasts [46] and induced genes involved in ribosome biogenesis and translation at least in *K. phaffii* [47].

Thus, a *K. phaffii* strain overexpressing the already spliced *HAC1*^i^ (Hac1 OE) and its empty vector control [46, 47] were used for OPP-labelling. Induction of UPR in the Hac1 OE strain was previously confirmed by microarray analysis [47]. In our experiment, when both strains were cultivated in glucose-excess conditions lower translational activity could be observed in the Hac1 OE strain compared to the empty vector control. However, in these conditions, both strains were growing at their maximum specific growth rates, which was μ = 0.29 h^− 1^ for Hac1 OE and μ = 0.38 h^− 1^ for the control. As translational activity and growth rate correlate, it is therefore hard to determine which factor (growth rate and/or UPR induction) was leading to reduced OPP-labelling in the Hac1 OE strain (Fig. 3c). Therefore, we cultivated both strains in glucose-limited conditions to equalize the growth rates. In these conditions, OPP-labelling showed the opposite behaviour for the Hac1 OE strain, indicating higher translational activity during UPR induction. This contradicts the assumption that translation is attenuated during UPR, but correlates well with increased transcription of secretion- and translation-related genes and increased productivity described in *K. phaffii* literature.

Furthermore, these results show how important growth rate is in translation activity measurement and that controls are indispensable for every OPP-labelling assay.

## Conclusion

We present a simple and non-invasive method for measuring global translational activity in the industrial yeast *K. phaffii*, which should be readily transferable to other yeast species. The established protocol of OPP-labelling in combination with Imipramine treatment can be used independently of the strain background. Furthermore, single cell analysis can provide important further information on single cell behaviour. Finally, our results indicate that using surfactants such as Imipramine, provides an advantageous alternative to knock out strains for increasing drug susceptibility, and should be applicable to many different areas of yeast research where drug or dye uptake are currently limiting.

## Methods

### Strain generation

*K. phaffii* CBS7435 and CBS2612 were provided by the Fungal Biodiversity Centre (Utrecht, Netherlands). The *ERG6* knockout strains, CBS7435 *erg6*Δ and CBS2612 *erg6*Δ were generated with the split-marker cassette method, through replacing the gene by a KanMX marker cassette [48]. The strain overexpressing the induced form of *HAC1*^i^ (GS115 pGAPHis+ HAC1) and its corresponding control strain transformed with the empty vector (GS115 pGAPHis+) were described before [46, 47, 49]. The *GCN2* gene was knocked out by using CRISPR/Cas9-based homology directed recombination [50].

### Cultivation conditions

Cultivations were done either in YP (20 g L^− 1^ peptone and 10 g L^− 1^ yeast extract) or in synthetic minimal media (ASMv6 [51]). For the cultivation of *gcn2*Δ, ASMv6 with less nitrogen was used, which contained only 2.50 g (NH_4_)_2_HPO_4_, 0.32 g (NH_4_)_2_SO_4_ and 8 mL NH_4_OH (25%).

For the minimum inhibitory concentration experiments, cultures were inoculated to an OD_600_ of 0.8 in YP media with 2% glucose (YPD) and different puromycin dihydrochloride concentrations. All cultivations were done for 48 h at 30 °C and 280 rpm.

For the growth curve experiments, overnight pre-cultures in YPD media were used for inoculation into 96-well microtiter plates at an OD_600_ of 0.5 with different concentrations of puromycin and/or surfactants added to either YP or ASMv6 media containing 2% glucose. The plates were incubated at 30 °C and 550 rpm in a microplate reader (Tecan Sunrise™) for 24 h while the OD_600_ was measured every 15 min. All cultivation conditions were measured in triplicate and subtracted by values of media incubated without cells.

To achieve glucose-limiting growth (GlucLim) conditions, 24-deep well plates were inoculated at an OD_600_ of 8 in ASMv6 supplemented with 50 g L^− 1^ polysaccharide (EnPump200, Enpresso) and 0.4% of glucose-releasing enzyme (Reagent A, Enpresso). Cultivation was done for different incubation times at 25 °C and 280 rpm. Growth rates were calculated according to a determined logarithmic curve comprising the correlation of growth rate (y-axis) and incubation time (x-axis) in these conditions: y = − 0.062ln(x) + 0.2413.

For glucose-excess (GlucExc) conditions, cells were inoculated in ASMv6 + 4% glucose at an OD_600_ of 0.2 in 24-deep well plates and incubated at 25 °C and 280 rpm for 20 h. The translation-inhibition control was cultivated under the same conditions with the exception that the media was supplemented with 20 g L^− 1^ hygromycin, and inoculation was done to an OD_600_ of 5.

Methanol-excess (MeOH 1 and MeOH 2) conditions were achieved by inoculating the cells to an OD_600_ of 10 in ASMv6 in 24- deep well plates as described above. Methanol was added 3 times to a final concentration of 1% per shot. For MeOH 1, shots were given after 0 h, 8 h and 20 h of cultivation, with subsequent OPP-labelling after 23 h. In case of the MeOH 2 feeding strategy, shots were given after 0 h, 16.5 h and 18 h of cultivation, with OPP-labelling after 19.5 h.

### Viability and metabolic activity testing (PI and 5-CFDA staining)

For the 5(6)-carboxy-2′,7′-dichlorofluorescein diacetate (5-CFDA) staining, cells were diluted to OD_600_ 0.4 in sodium citrate buffer (50 mM pH = 4.5) and treated with different concentrations of surfactants (Imipramine, LiCl/DTT, PEG4000, Pluronic® PE 6100, Triton™ X-100 or Tween®20). After adding 10 mg L^− 1^ 5-CFDA (10 g L^− 1^ stock dissolved in DMSO), the suspension was incubated for 30 min at RT. Subsequently, propidium iodide (PI) was added to a final concentration of 2 μM and the samples were measured by flow cytometry. As controls, non-viable (heat-inactivated) cells, non-treated *K. phaffii* (negative control), and non-treated *erg6*Δ (positive control) were measured unstained, single stained and double stained.

### OPP-labelling

For the OPP assay, an “incubation solution”, consisting of ASMv6 supplemented with 0.30 g L^− 1^ (0.6 mM) OPP (dissolved in 10% DMSO and PBS (2 mM KH_2_PO_4_, 10 mM Na_2_HPO_4_*2H_2_O, 2.7 mM g KCl, 8 mM NaCl, pH 7.4)) and Imipramine (1.5 g L^− 1^ if not mentioned otherwise) was prepared. For the no-OPP control (included in every independent measurement) ddH_2_O was substituted for OPP. Cells were harvested and immediately diluted to OD_600_ 0.4 into 90 μL “incubation solution” in a 96-well microtiter plate and incubated for 2 h at 25 °C and 1200 rpm. Subsequently, the suspension was transferred into ice-cold Eppendorf tubes and the cells pelleted by centrifugation at 16,000 g for 5 min at 4 °C. After discarding the supernatant and washing the cells with 120 μL PBS, the cells were fixed with 1 mL of ice-cold 70% ethanol. These fixed samples can be stored between 1 day and at least 2 weeks at 4 °C.

### Click chemistry

The click chemistry reaction was done as described in Nagelreiter et al. [14]. Briefly, the fixed samples were harvested by centrifugation at 16,000 g and 4 °C for 5 min. The resulting pellet was dissolved in 100 μL “Click Chemistry Buffer” (115 mM Tris/HCl pH = 8.5, 0.1% Triton X-100), transferred to a 96-well microtiter plate, harvested and incubated in 150 μL “Click Chemistry Mix” (101 mM Click-it Click Chemistry Buffer, 1.9 mM CuSO4, 1.9 mg mL^− 1^ ascorbic acid, 20 μM Alexa Fluor™ 488 azide (AF488; Invitrogen)) for 30 min at RT. Then, the cells were harvested as described before, washed once in 150 μL PBS and resuspended in 150 μL fresh PBS.

### Flow cytometry

Fluorescence intensities of stained cells were analysed by flow cytometry (CytoFlex, Beckmann Coulter). The 488 nm laser was used for excitation of all samples. For emission, the optical filter 690/50 BP was used for PI, and 525/40 BP for AF488 and 5-CFDA. Forty thousand events were measured for each sample and gates set appropriately to exclude cell debris. For numerical data analysis, the geometric mean of each sample subtracted by a negative control (e.g. no-OPP) was used. For relating translation activity to cell size, the fluorescence signal was divided by the forward scatter (FSC) to the power of 1.5 [52]. The data was analysed with Kaluza Analysis 2.1.

## Supplementary Information

**Additional file 1: Supplementary Figures.** Figures showing the preliminary minimal inhibitory concentration experiment (Fig. S1), 5-CFDA flow cytometry histograms (Fig. S2), 5-CFDA and PI flow cytometry density plots (Fig. S3), growth curve assays determining Imipramine and puromycin concentrations (Fig. S4) and plots illustrating the relationship between cell size and global translation activity (Fig. S5). 

## Data Availability

The datasets used and/or analysed during the current study are available from the corresponding author on reasonable request.

## Abbreviations

5-CFDA: 5(6)-carboxy-2′,7′-dichlorofluorescein diacetate
AF488: Alexa Fluor™ 488 azide
DMSO: Dimethyl sulfoxide
DTT: Dithiothreitol
EPP: *S. cerevisiae erg6*Δ, *pdr1*Δ *pdr3*Δ
FSC: Forward scatter
OE: Overexpression
OPP: O-propargyl-puromycin
PI: Propidium iodide
UPR: Unfolded protein response
