# Supplementary material for: Treatment with surfactants enables quantification of translational activity by O-propargyl-puromycin labelling in yeast
Source: BMC Microbiol. 2021 Apr 20;21:120. doi: 10.1186/s12866-021-02185-3 (PMC8056590; doi:10.1186/s12866-021-02185-3)
Supplement: Supplementary file 1 — Additional file 1: Supplementary Figures. Figures showing the preliminary minimal inhibitory concentration experiment (Fig. S1), 5-CFDA flow cytometry histograms (Fig. S2), 5-CFDA and PI flow cytometry density plots (Fig. S3), growth curve assays determining Imipramine and puromycin concentrations (Fig. S4) and plots illustrating the relationship between cell size and global translation activity (Fig. S5). [file 12866_2021_2185_MOESM1_ESM.pdf]

Supporting Information
for

Treatment with surfactants enables quantification of translational activity by O-propargyl-puromycin labelling in yeast

Jennifer Staudacher[1,2], Corinna Rebnegger[1,2], Brigitte Gasser[1,2*]

[1] Christian Doppler Laboratory for Growth-decoupled Protein Production in Yeast, Vienna, Austria

[2] Department of Biotechnology, University of Natural Resources and Life Sciences (BOKU), Vienna, Austria

*Corresponding author:

Brigitte Gasser, Institute of Microbiology and Microbial Biotechnology, Department of Biotechnology, BOKU University of Natural Resources and Life Sciences

Muthgasse 18, 1190 Vienna, Austria

brigitte.gasser@boku.ac.at

Brigitte Gasser, http://orcid.org/0000-0003-2881-6370

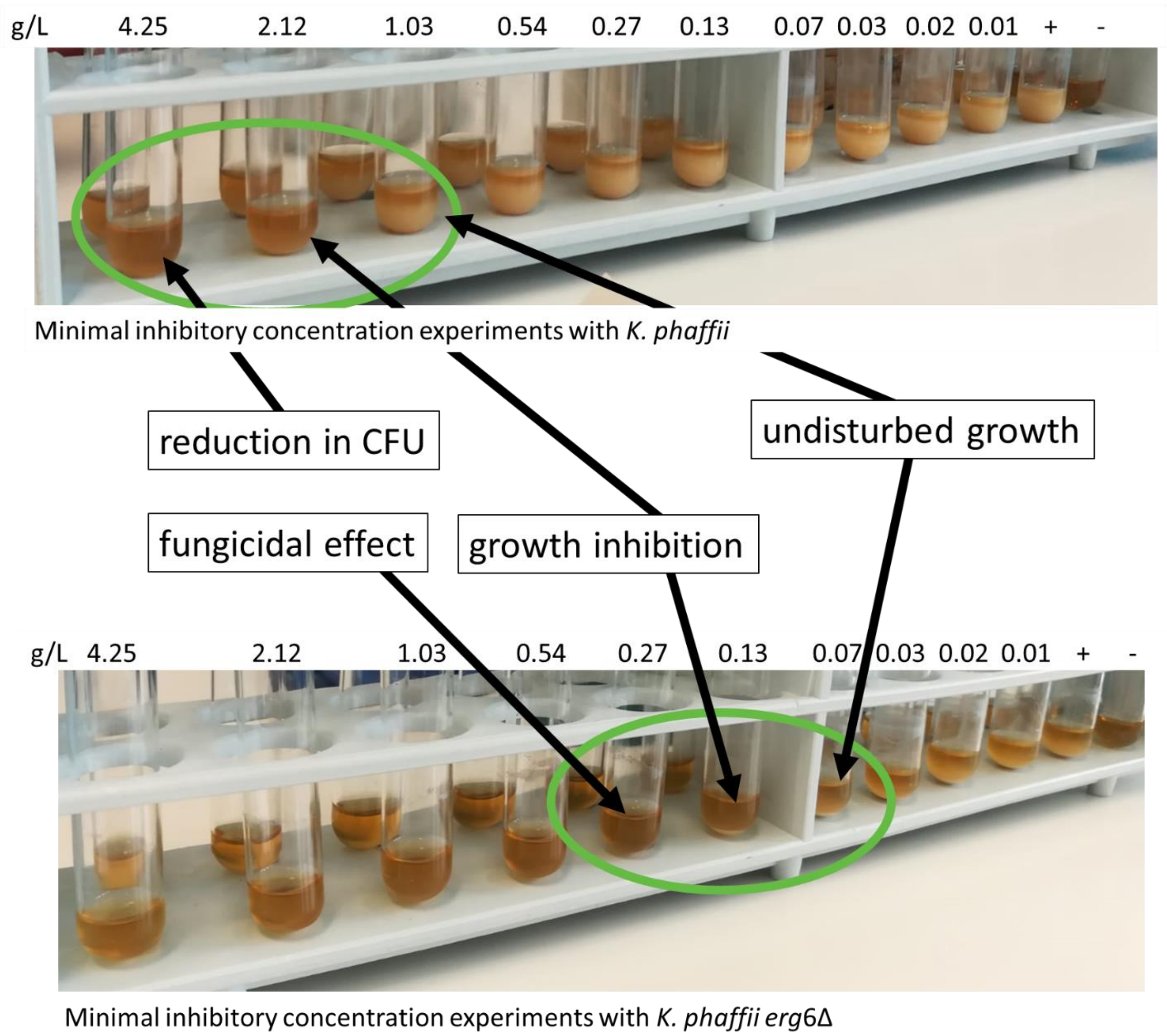

Supplementary Figure S1. Minimal inhibitory puromycin concentration of *K. phaffii*. The *K. phaffii* control and erg6Δ mutant were cultivated for 48 h in YPD in the presence of different concentrations of puromycin ranging from 0.01 to 4.25 g/L. For the positive control (+) cells were incubated without puromycin addition, for the negative control (-) the media was incubated without cells and puromycin. The lower picture shows the susceptibility of *K. phaffii erg6*Δ, the upper shows the parental *K. phaffii* strain.

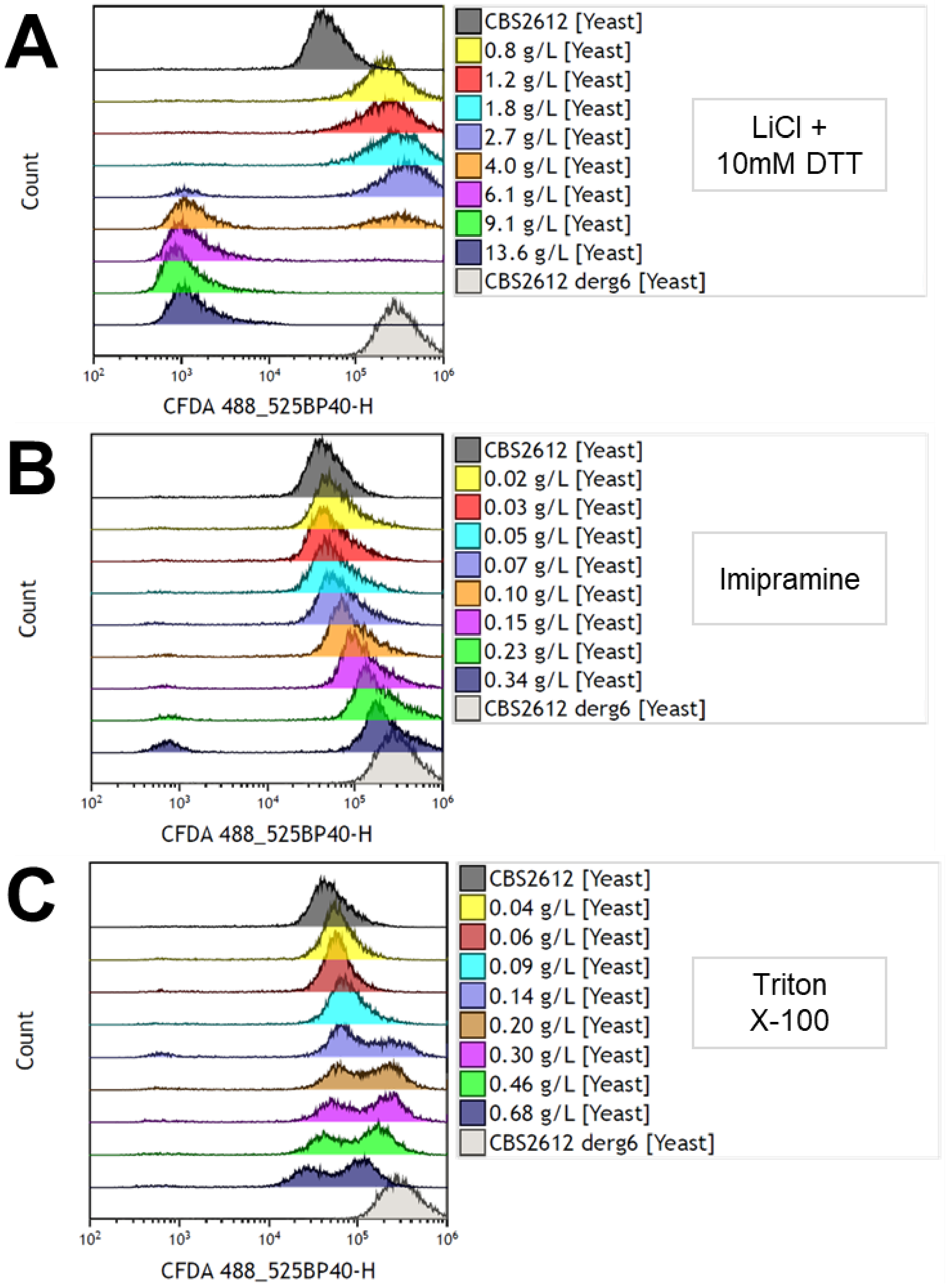

Supplementary Figure S2. 5-CFDA signal histograms from flow cytometry after incubation of *K. phaffii* CBS2612 with different concentrations of A) LiCl with always 10 mM DTT, B) Imipramine and C) Triton X-100. Untreated cells (denoted as CBS2612) were used as negative control, untreated *erg6*Δ cells (denoted as CBS2612 derg6) were used as positive control. The yeast population was gated to exclude debris in all samples.

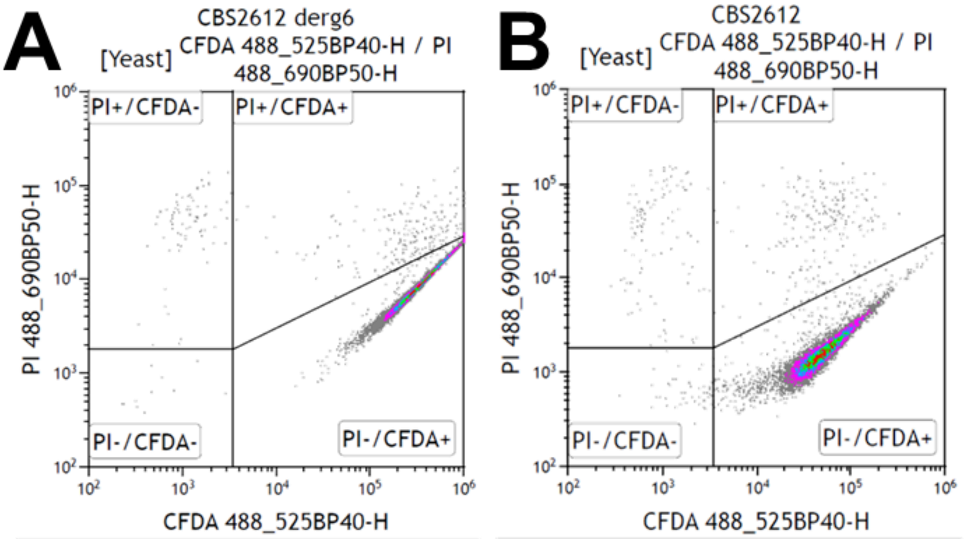

Gate	%Gated
All	100.00
PI-/CFDA-	0.11
PI-/CFDA+	95.81
PI+/CFDA-	0.79
PI+/CFDA+	3.29

Gate	%Gated
All	100.00
PI-/CFDA-	0.52
PI-/CFDA+	96.06
PI+/CFDA-	1.11
PI+/CFDA+	2.31

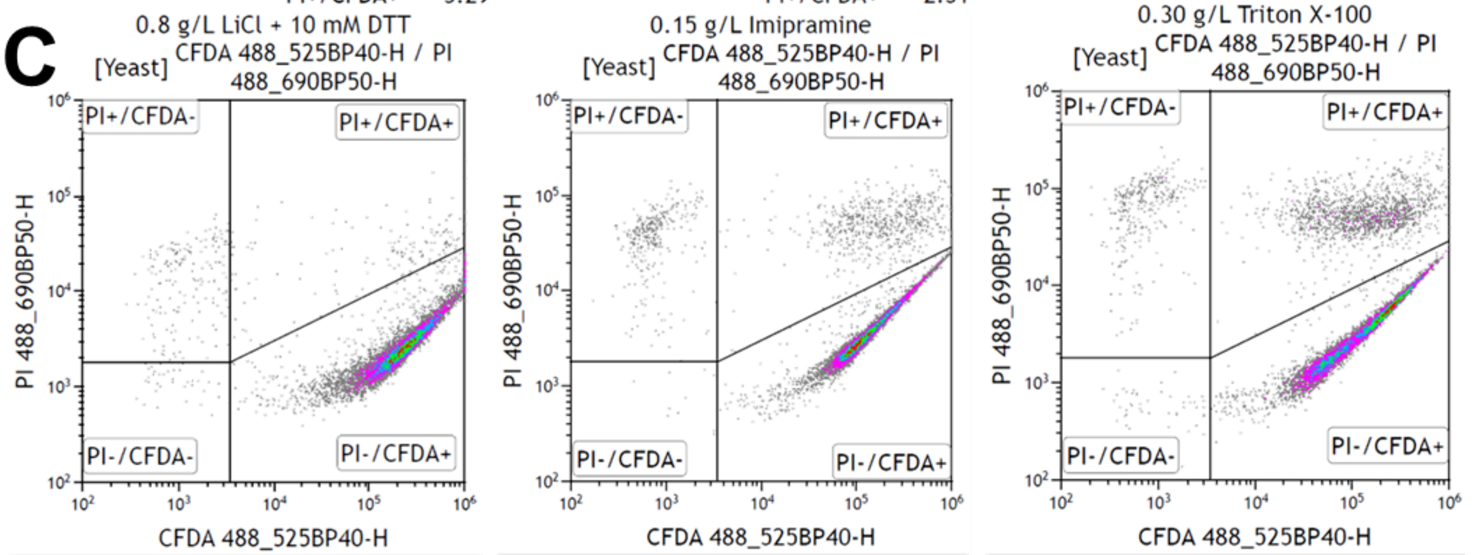

Gate	%Gated
All	100.00
PI-/CFDA-	0.41
PI-/CFDA+	95.76
PI+/CFDA-	2.01
PI+/CFDA+	1.82

Gate	%Gated
All	100.00
PI-/CFDA-	0.10
PI-/CFDA+	89.50
PI+/CFDA-	3.49
PI+/CFDA+	6.91

Gate	%Gated
All	100.00
PI-/CFDA-	0.49
PI-/CFDA+	80.99
PI+/CFDA-	3.20
PI+/CFDA+	15.31

Supplementary Figure S3. Fluorescence signal density plots from flow cytometry after 5-CFDA (x-axis) and PI (y-axis) staining of A) *K. phaffii* CBS2612 (used as negative control) and B) *K. phaffii* CBS2612 *erg6*Δ cells (used as positive control). C) Staining of *K. phaffii* CBS2612 treated with the surfactant concentrations chosen for further testing. These chosen concentrations showed the best compromise between highest obtained 5-CFDA signal and the least PI positive cells possible. The yeast population was gated to exclude debris in all samples.

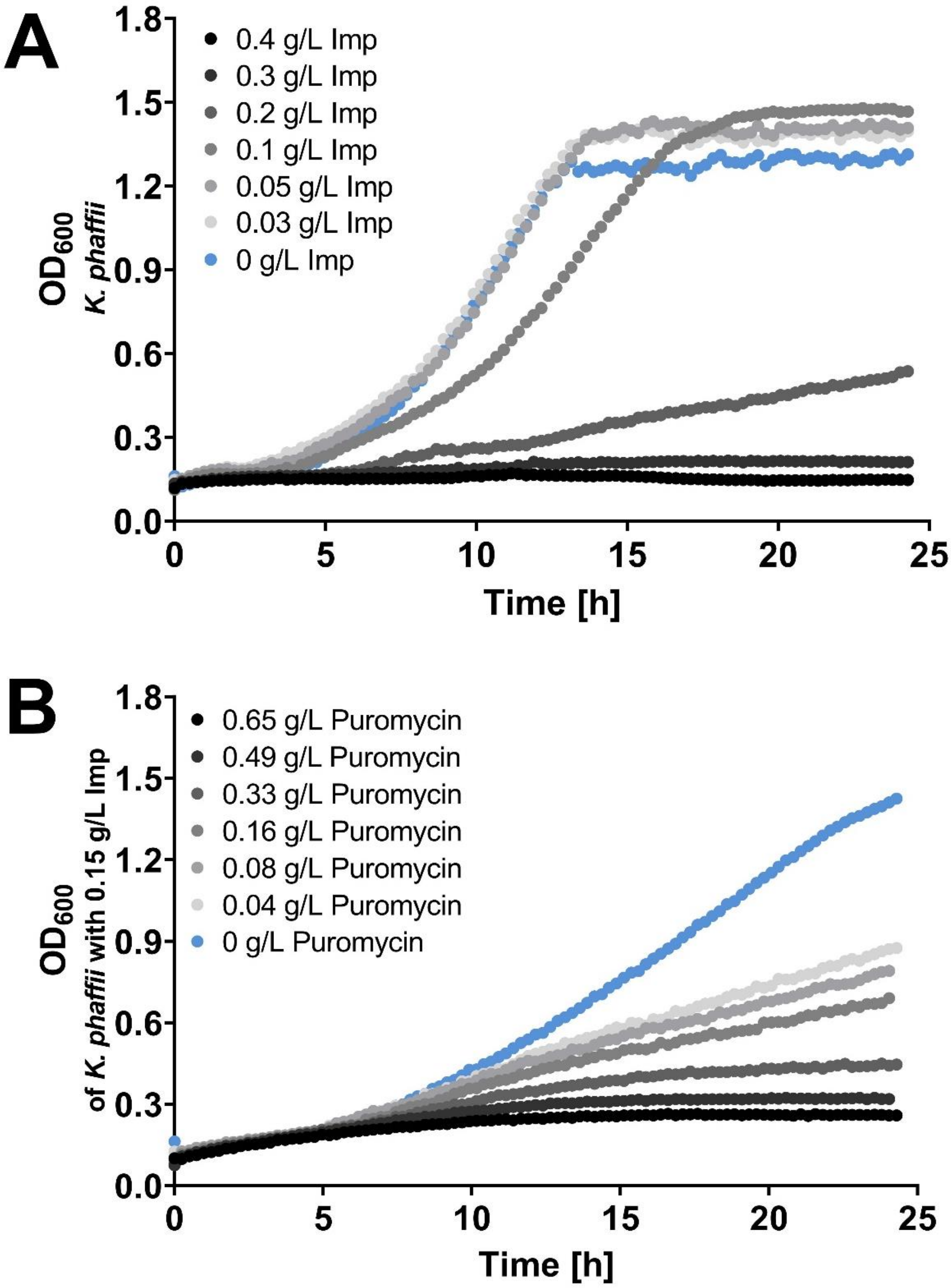

Supplementary Figure S4. Growth curve assays for determining Imipramine and corresponding dose-dependent puromycin effect. The OD_{600} was measured every 15 min in a microplate reader for 24 h at 30°C and 550 rpm. All growth curves were measured in biological triplicates. As controls, untreated cells were included in triplicate on each assay plate. A) *K. phaffii* CBS2612 treated with different Imipramine (Imp) concentrations. After further testing, an Imipramine concentration of 0.15 g/L was chosen as a compromise between most effect on puromycin susceptibility and the least amount of growth impairment. B) *K. phaffii* CBS2612 treated with 0.15 g/L Imipramine and different puromycin concentrations.

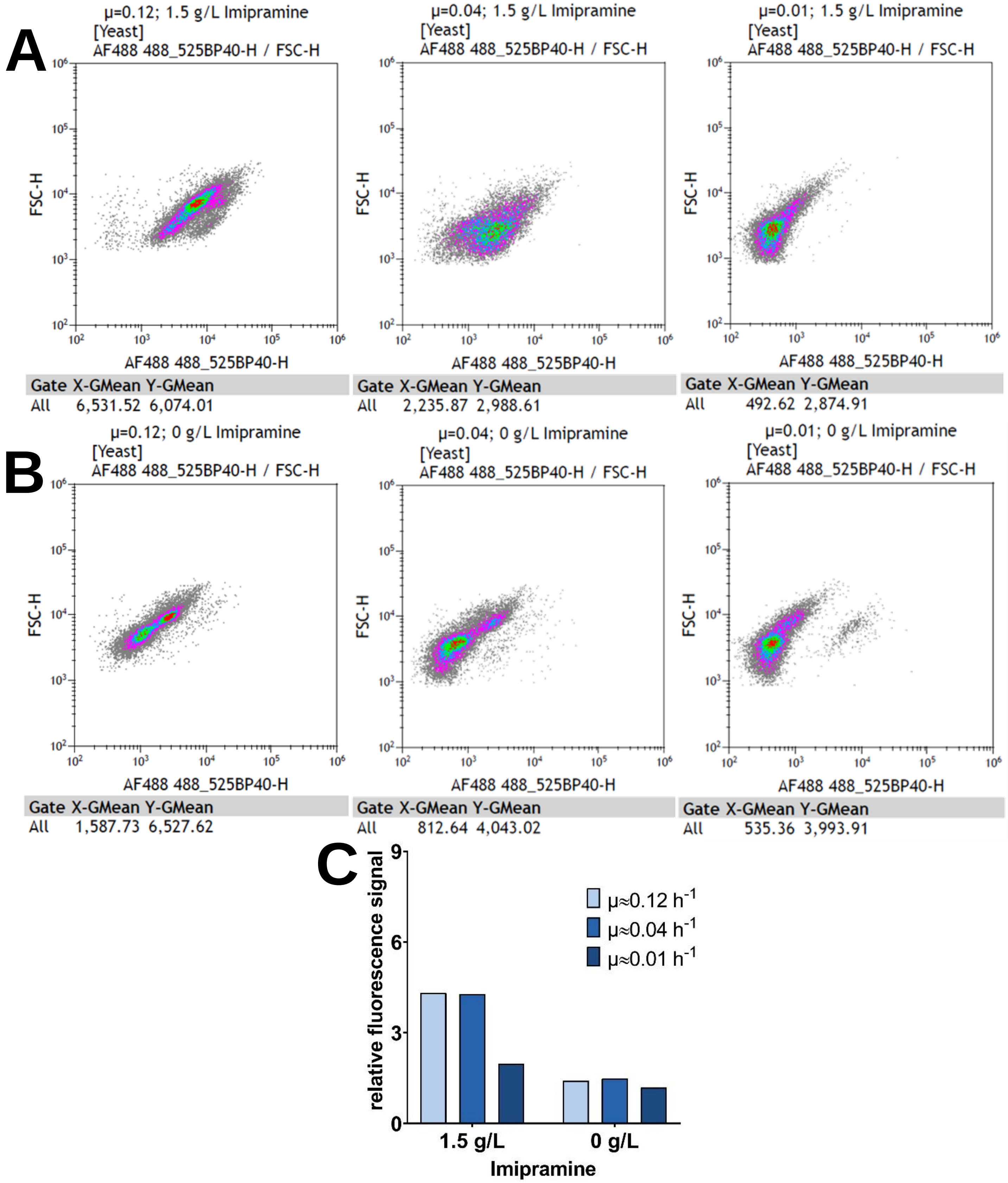

Supplementary Figure S5. Translation activity and cell size relative to growth rate of *K. phaffii* CBS2612 in glucose-limit conditions. A) and B) show density plots where translation activity (AF488 signal) was plotted on the x-axis and cell size (FSC signal) on the y-axis. The OPP-labelling results are shown when A) 1.5 g /L Imipramine was used or when B) no Imipramine was used during the OPP-assay. The growth rate (μ) decreases from the left to the right density plots. The automatic geometric mean calculation is additionally shown for both signals. The yeast population was gated to exclude debris for each sample. C) shows the calculated translation activity relative to cell size, based on the FSC, for all the samples. The shown values were also related to the no-OPP control.
